# Neuroprotective Role of GLP-1 Analog for Retinal Ganglion Cells via PINK1/Parkin-Mediated Mitophagy in Diabetic Retinopathy

**DOI:** 10.3389/fphar.2020.589114

**Published:** 2021-02-12

**Authors:** Huan-ran Zhou, Xue-fei Ma, Wen-jian Lin, Ming Hao, Xin-yang Yu, Hong-xue Li, Cheng-ye Xu, Hong-yu Kuang

**Affiliations:** Department of Endocrinology, The First Affiliated Hospital of Harbin Medical University, Harbin, China

**Keywords:** GLP-1, GLP-1 analog, diabetic retinopathy, mitophagy, retinal ganglion cell

## Abstract

GLP-1 analogs have been widely used to treat patients with type 2 diabetes in recent years and studies have found that GLP-1 analogs have multiple organ benefits. However, the role of GLP-1 analogs in diabetic retinopathy (DR), a common complication of diabetes mellitus (DM), remains controversial. Retinal ganglion cells (RGCs) are the only afferent neurons responsible for transmitting visual information to the visual center and are vulnerable in the early stage of DR. Protection of RGC is vital for visual function. The incretin glucagon-like peptide-1 (GLP-1), which is secreted by L-cells after food ingestion, could lower blood glucose level through stimulating the release of insulin. In the present study, we evaluated the effects of GLP-1 analog on RGCs both *in vitro* and *in vivo*. We established diabetic rat models *in vivo* and applied an RGC-5 cell line *in vitro*. The results showed that in high glucose conditions, GLP-1 analog alleviated the damage of RGCs. In addition, GLP-1 analog prevented mitophagy through the PINK1/Parkin pathway. Here we demonstrated the neuroprotective effect of GLP-1 analog, which may be beneficial for retinal function, and we further elucidated a novel mechanism in GLP-1 analog-regulated protection of the retina. These findings may expand the multi-organ benefits of GLP-1 analogs and provide new insights for the prevention of DR.

## Introduction

Diabetic retinopathy (DR) is one of the main complications of diabetes mellitus (DM) and is the major cause of blindness in working-age adults, which seriously reduces quality of life and consumes a lot of health resources (Wong et al., 2016). Attention should be paid to DR in the early stage of DM. The retina is recognized as a part of the central nervous system during neurodevelopment (Stern and Temple, 2011). Vascular lesions in DR have been extensively studied for years, and neurological changes are arousing more and more concerns. Antonetti et al. (2012) demonstrated that diabetes-related retinal dysfunction is not confined to microangiopathy but is associated with neurovascular unit lesions. The protection of retinal neurocytes may also be beneficial for microvasculature. Wolter (1961) indicated that the first change in retina of DM was the degeneration of retinal neurons, which may cause vascular damage. More interestingly, Barber et al. (1998) found the density of RGCs reduced in diabetic rats compared with the control group. RGCs, which are responsible for the entire retinal signals’ output to the brain of vertebrates, with their long axons together composed the inner layer of the retina (Baden et al., 2016). Although the mechanisms of RGCs injury remain unclear, the articles above have suggested the importance of RGCs protection.

Mitochondria respond to changes in the metabolic environment through altering morphology and function. A high glucose environment, such as hyperglycemia, induces destruction of mitochondria, excessive production of reactive oxygen species (ROS), and even residues of damaged mitochondrial DNA. The oversupply of energy induces injury of mitochondria and causes a potential toxic conditions of cells (Picard and Turnbull, 2013). RGCs are vulnerable to mitochondrial damage as the enrichment of mitochondria colonization, and have a rapid metabolism and high energy dependence (Carelli et al., 2004). Damaged mitochondria are digested through mitophagy (Kim et al., 2007), which is a specialized form of autophagy and is one of the mitochondrial regulatory mechanisms. However, excessive mitophagy induces cell death (Shi et al., 2014). Mitophagy plays a noticeable role in neurodegenerative disorders (Palikaras and Tavernarakis, 2012), such as Alzheimer’s (Ye et al., 2015), Parkinson’s (Ryan et al., 2015), and Huntington’s diseases (Khalil et al., 2015). But the effect of mitophagy on RGCs is unclear and needs to be further explored. The regulation of mitophagy and possible pathways may be potential therapeutic targets for RGCs protection in DR.

Glucagon-like peptide-1 (GLP-1), which is secreted by intestinal L cells, reduces the value of blood glucose by promoting the secretion of insulin after a meal (Meier, 2012). In recent years, GLP-1 analogs have been proven to provide multiple organ benefits and gradually become a commonly used type of hypoglycemic drug in clinic. GLP-1 analogs could reduce the risks of microvascular complications of DM and are recommended for patients with cardiovascular disease or diabetic kidney disease in the American Diabetes Association (ADA) “Standards of Medical Care in Diabetes-2020” (American Diabetes Association, 2020a; American Diabetes Association, 2020b). Studies have also found that GLP-1 analog has neuroprotective effects (Pang et al., 2018). Therefore, we consider that GLP-1 analog may provide an additive benefit to DR. However, the effects of GLP-1 analog on RGCs and its possible mechanisms are still largely unknown and remain to be investigated.

In the present study, we aimed to demonstrate the effect of GLP-1 analog on RGCs and the potential mechanisms both *in vivo* and *in vitro*. We found GLP-1 analog protected RGCs from a high glucose environment and investigated the benefits from the inhibition of mitophagy via the PINK1/Parkin pathway. Based on these results, GLP-1 analog may provide advantageous effects for the retina in the early stage of DR.

## Methods

### Cell Culture and Treatment

RGC-5 cells were gained from the laboratory of Fudan University and cultured with DMEM containing 10% fetal bovine serum (FBS) and 1% antibiotics (100 U/mL penicillin and 100 mg/ml streptomycin) at 37°C in a 5% CO_2_ incubator. Cells during the logarithmic phase were treated with different reagents.

### Cell Viability Assay

CCK-8 detection kit (Dojindo Laboratories, CK04) was used to test cell viability. RGC-5 cells were incubated with different concentrations of glucose (25, 40, 65, 80, 95, and 110 nmol/L). Then cells were co-treated with normal glucose (25 mmol/L) (Ji et al., 2011) or high glucose (65 mmol/L) and different doses of GLP-1 analog (liraglutide) (1, 10, 50, 100, 500, and 1000 nmol/L) for 24 h. Then CCK-8 reagent was used to treat cells and the plate was placed at 37°C for 1 h. Absorbance measurements at a wavelength of 450 nm were counted by a monochromator microplate reader.

### Western Blotting

8–12% SDS–PAGE was used to resolve equivalent amounts of protein and was then transferred to a nitrocellulose filter membrane. The membranes were incubated with primary antibodies LC3A/B (1:1000 dilution, #4108, Cell Signaling Technology, Massachusetts, United States), p62 (1:2000 dilution, ab56416, Abcam, Massachusetts, United States), PINK1 (1:1000 dilution, 23274-1-AP, Proteintech Group, Illinois, United States), Parkin (1:1000 dilution, ab15954, Abcam, Massachusetts, United States), and GAPDH (1:2000 dilution, #2118, Cell Signaling Technology, Massachusetts, United States) overnight at 4°C after blocking the membranes with 5% nonfat milk for 1 h, and then incubated with the appropriate IRDye-conjugated secondary antibody (1:10000 dilution, IRDye 800 CW goat anti-rabbit or IRDye 680RD goat anti-mouse, LI-COR Biosciences, Nebraska, United States) and imaged using the LI-COR Odyssey. Image Studio 5.2 software was used for density analysis.

### Transmission Electron Microscopy

Cells and tissues were fixed with 1% OsO_4_ and washed after fixing in 2.5% glutaraldehyde in a 0.1 M phosphate. Then cells and tissues were dehydrated with graded alcohol and embedded in epoxy resin. Ultrathin sections stained with uranyl acetate and lead citrate were put on copper grids and imaged with a transmission electron microscope (JEOL, Tokyo, Japan).

### Immunofluorescence Staining

4% paraformaldehyde was used for fixing cells for 30 min after treatment. 0.5% Triton X-100 was used to permeabilize cells for 20 min. Then cells were blocked in 1% BSA at room temperature for 1 h. Cells were treated with the primary antibody overnight at 4°C and followed by secondary antibodies (ZSGB-BIO) in the dark at room temperature for 1 h. DAPI (Beyotime) was used to stain nuclei. Images were captured with a fluorescence microscope (EVOS FL Auto, Life Technologies).

### Measurement of Reactive Oxygen Species Generation

ROS levels were detected by the fluorescent probe 2′,7′-dichlorofluorescin diacetate (DCFH-DA, Beyotime). Cells were treated with DCFH-DA in the dark at 37°C for 25 min and serum-free DMEM was used to rinse cells three times. A fluorescence microscope (EVOS FL Auto, Life Technologies) was used to observe the fluorescence intensity.

### Measurement of Mitochondrial Membrane Potential

MMP was assayed by the fluorescent probe 5,5′,6,6′-Tetrachloro-1,1′,3,3′-tetraethyl-imidacarbocyanineiodide (JC-1, Beyotime). After being treated with JC-1 solution (10 mg/ml) for 25 min at 37°C, cells were washed with buffer (precooled at 4°C). A fluorescence microscope (EVOS FL Auto, Life Technologies) was used to detect the fluorescence intensity.

### Immunocytochemistry Staining

Cells were grown on autoclaved uncoated glass coverslips in 6-well plates. The cells were fixed in 4% paraformaldehyde for 20 min and blocked at room temperature for 30 min with 5% normal goat serum (in PBS) after treatment. Then, cells were incubated overnight at 4°C by using primary antibodies LC3A/B (1:1000 dilution, #4108, Cell Signaling Technology, Massachusetts, United States), p62 (1:2000 dilution, ab56416, Abcam, Massachusetts, United States), PINK1 (1:1000 dilution, 23274-1-AP, Proteintech Group, Illinois, United States), and Parkin (1:1000 dilution, ab15954, Abcam, Massachusetts, United States). After washing with PBS, secondary antibodies biotinylated anti-mouse IgG (1:250 dilution, BA-9200, Vector Laboratories, California, United States) and biotinylated anti-rabbit IgG (1:250 dilution, BA-1000, Vector Laboratories, California, United States) were added at 37°C for 20 min and incubated with avidin-biotin-peroxidase reagents (SABC) at 37°C for 20 min. The cells were washed twice for 3min each and treated in 0.1% 3,3′-diaminobenzidine (DAB) for 2–5 min; they were then dehydrated in a graded series of ethanol and counterstained for 1 min with haematoxylin. All samples were processed under the same conditions.

### Animal Studies

Male Sprague-Dawley (SD) rats (6 weeks old, weighing 160–200 g) were obtained from the Experimental Animal Center at the Second Affiliated Hospital of Harbin Medical University. The rats were divided into a normal diet (12% fat, 21% protein, and 65% carbohydrate) group (control group, *n* = 8) and a high-fat diet (43% fat, 17% protein, and 40% carbohydrate) group (HFD group, *n* = 16). 8 weeks later, the rats of the HFD group were intraperitoneally injected with 30 mg/kg of Streptozotocin (STZ, Sigma, St. Louis, MO, United States) after fasting for 12 h, and citrate buffer was injected into rats of the control group. Rats with fasting blood glucose concentrations ≥11.1 mmol/L for three continuous days were considered as successful diabetes models (Srinivasan et al., 2005). Then the rats of the HFD group were randomly divided into the DM group (*n* = 8) and GLP-1 analog treatment group (*n* = 8). Rats of the GLP-1 analog treatment group were treated with liraglutide (Novo Nordisk) for 8 weeks at a dose of 0.2 mg/kg/d by subcutaneous injection, while rats of the control group and DM group were treated with equal levels of saline. The dose was based on the guidelines provided by the Center for Drug Evaluation and Research (CDER) at the Food and Drug Administration (Reagan-Shaw et al., 2008) and its effect on diabetics (Peterson and Pollom, 2010). Hyperglycemia induced by STZ usually occurs within 2 weeks 4–5 weeks after the onset of hyperglycemia, an increase in the number of astrocytes and glial proliferation could be observed. The RGCs began to deplete at 6 weeks. The inner and outer nuclear layers of retina become thinner at 10 weeks. Neovascularization formed at 16 weeks, and there were acellular capillaries and pericyte ghosts after 6 months (Malek et al., 2018). In order to study RGCs better, we chose 8 weeks as the end of the observation. The study was approved by the Ethics Committee of The First Affiliated Hospital of Harbin Medical University and conformed to the principles of the Declaration of Helsinki. All animal procedures were conducted in accordance with the National Institutes of Health’s Guide for the Care and Use of Laboratory Animals (NIH Publications number 8023, revised 1978).

### Immunohistochemistry Staining

Tissue sections were deparaffinized with xylene and rehydrated with ethanol, followed by antigen retrieval. Optimal serum was used to block tissue sections. Primary antibodies LC3A/B (1:1000 dilution, #4108, Cell Signaling Technology, Massachusetts, United States), p62 (1:2000 dilution, ab56416, Abcam, Massachusetts, United States), PINK1 (1:1000 dilution, 23274-1-AP, Proteintech Group, Illinois, United States), and Parkin (1:1000 dilution, ab15954, Abcam, Massachusetts, United States) were used to incubate tissue sections overnight at 4°C. Then, tissue sections were incubated with secondary antibody biotinylated anti-mouse IgG (1:250 dilution, BA-9200, Vector Laboratories, California, United States) and biotinylated anti-rabbit IgG (1:250 dilution, BA-1000, Vector Laboratories, California, United States) at room temperature for 1 h, followed by the Vectastain Elite ABC reagent (Vector lab) for 30 min. The tissue sections were counterstained with hematoxylin (Solaria) after the peroxidase reaction with diaminobenzidine (DAB Kit; Solaria). Positive staining was indicated by brown deposits. Mean optical density was analyzed using Image-Pro Plus v6.0 software as follows. Images was opened in Image Pro Plus software, then Measure was selected in the menu bar; Calibration in the Measure menu was selected and Intensity was chosen, followed by New and Std. For optical density, options was selected and the Optical Density Calibration box was entered. In the box, image of incident level was clicked, then the bright white space of image was clicked. OK was selected and the user was returned to the Optical Density Calibration box. Next, the Minimize button was selected and the user was returned to the image window. In the window, Magic Wand was clicked and ganglion cell layer was selected; Count/Size in the Measure menu was clicked and the Count/Size dialogue box was opened. Measure and then Select Measurements was selected; Area and IOD were clicked and then settings were saved. Select colors and then select HIS were selected, and then the options set up and saved. Data collector was opened, then area and IOD were clicked. Count and then Collect Now were selected. IOD and area were collected separately, while mean optical density was calculated by IOD/area. Instructions are available at https://mediacy.com.cn/imageproplus/learn/media.

### Statistical Analysis

For western blotting and IHC staining, the representative images were shown. Each of these experiments was independently repeated 3 times. Results are reported as mean ± standard error of the mean (S.E.M.) of at least three independent experiments. Statistical analysis was performed using GraphPad Prism 5 software. Comparisons were performed using unpaired one-way ANOVA (*p < 0.05, **p < 0.01, ***p < 0.001) as indicated in individual figures. A *p*-value < 0.05 was considered statistically significant. The investigators were not blinded to allocation during experiments and outcome assessment.

## Results

### Liraglutide Attenuated Retinal Ganglion Cells Damages Caused by High Glucose *In Vitro*

**FIGURE 1:**
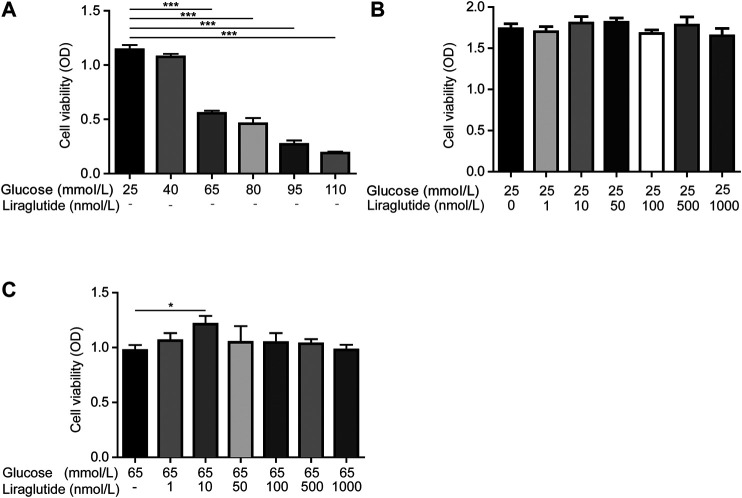
Liraglutide mitigated high glucose-induced RGCs injuries *in vitro*. As shown in the figure, RGCs were treated with different concentrations of glucose (25, 40, 65, 80, 95, 110 mmol/L) and different doses of liraglutide (0, 1, 10, 50, 100, 500, and 1000 nmol/L) for 24 h **(A–C)** Cell viability was detected with CCK-8 assay kits. Values are presented as mean ± SEM. *n* = 3 independent experiments. *p < 0.05, ***p < 0.001.

We created diabetic conditions using high glucose *in vitro*. RGC-5 cells were treated with DMEM containing different concentrations of glucose. Cell viability was measured by CCK-8 detection kit. The results showed that high glucose decreased the viability of RGCs compared with normal glucose (25 mmol/L), and it was statistically significant when the concentration of glucose was up to 65 mmol/L (Figure 1A). Then we co-incubated cells with normal glucose and different doses of liraglutide in order to detect the effect of liraglutide in normal glucose conditions. The results showed that in a normal glucose environment, liraglutide showed no significant effect on cell viability and cells could tolerate the concentration of liraglutide up to 1000 nmol/L (Figure 1B). Next, cells were treated with high glucose (65 mmol/L) and dissimilar doses of liraglutide. We found that 10 nmol/L liraglutide significantly improved cell viability (Figure 1C). Based on the above results, we selected 25 mmol/L glucose as the control group *in vitro*. 65 mmol/L high glucose and 10 nmol/L liraglutide were considered as reagents for the following experiments *in vitro*.

### Liraglutide Reduced Mitochondrial Damage in Retinal Ganglion Cells Caused by High Glucose *In Vitro*

**FIGURE 2:**
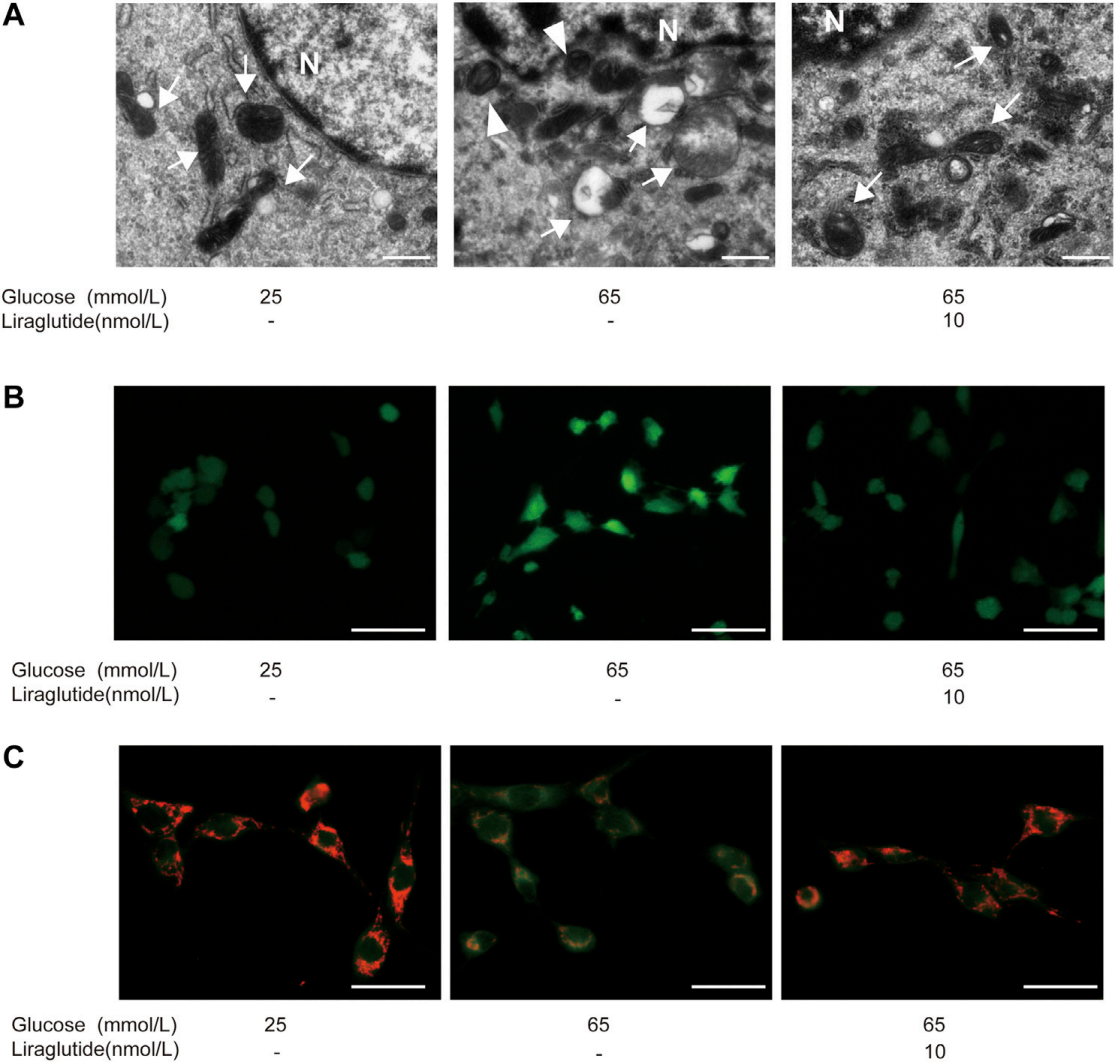
Liraglutide alleviated high glucose-induced mitochondrial damage *in vitro*. RGCs were respectively treated with normal glucose (25 mmol/L), high glucose (65 mmol/L), high glucose (65 mmol/L) and liraglutide (10 nmol/L) for 24 h. **(A)** Morphology changes of RGCs were observed by TEM. Scale bar: 2.0 μm. ►, autophagosomes; mitochondria; N, nucleus. **(B)** The generation of reactive oxygen species (ROS) in RGCs was detected by fluorescent probes DCFH-DA. Scale bar: 400 μm. **(C)** Mitochondrial membrane potential (MMP) was detected by fluorescent probes JC-1. Scale bar: 400 μm.

We observed the ultrastructure of RGCs by TEM. The results showed that mitochondrial cristae in cells treated with high glucose became indistinguishable, shortened, and disappeared. Furthermore, mitochondria were swollen and exhibited vacuole-like changes. After being treated with liraglutide, the structure of mitochondrial cristae was clear and the degree of mitochondrial swelling was alleviated (Figure 2A). The above results suggested that high glucose induced mitochondrial damage and liraglutide could protect mitochondria from a high glucose environment. Next, immunofluorescence was used to detect the production of ROS. The results showed that cells in a high glucose environment had a higher level of ROS compared to control groups and liraglutide markedly decreased high glucose-induced ROS augmentation (Figure 2B). Moreover, we detected the change of MMP, which could reflect the status of the mitochondria (Zhang et al., 2015). The mitochondria in the control group mainly emitted red fluorescence. After the incubation of high glucose, we found MMP of cells declined, which was represented by the emission of green fluorescence. However, liraglutide treatment increased the production of red fluorescence in mitochondria and enhanced MMP of cells (Figure 2C).

### Liraglutide Alleviated Retinal Ganglion Cells Injuries by Weakening Mitophagy *In Vitro*

To explore whether mitophagy played a role in high glucose-induced RGCs injury, we used TEM to observe autophagosome in cells directly. The results showed that high glucose enhanced autophagosome formation, while there was almost no typical autophagosome after the treatment of liraglutide (Figure 2A).

**FIGURE 3:**
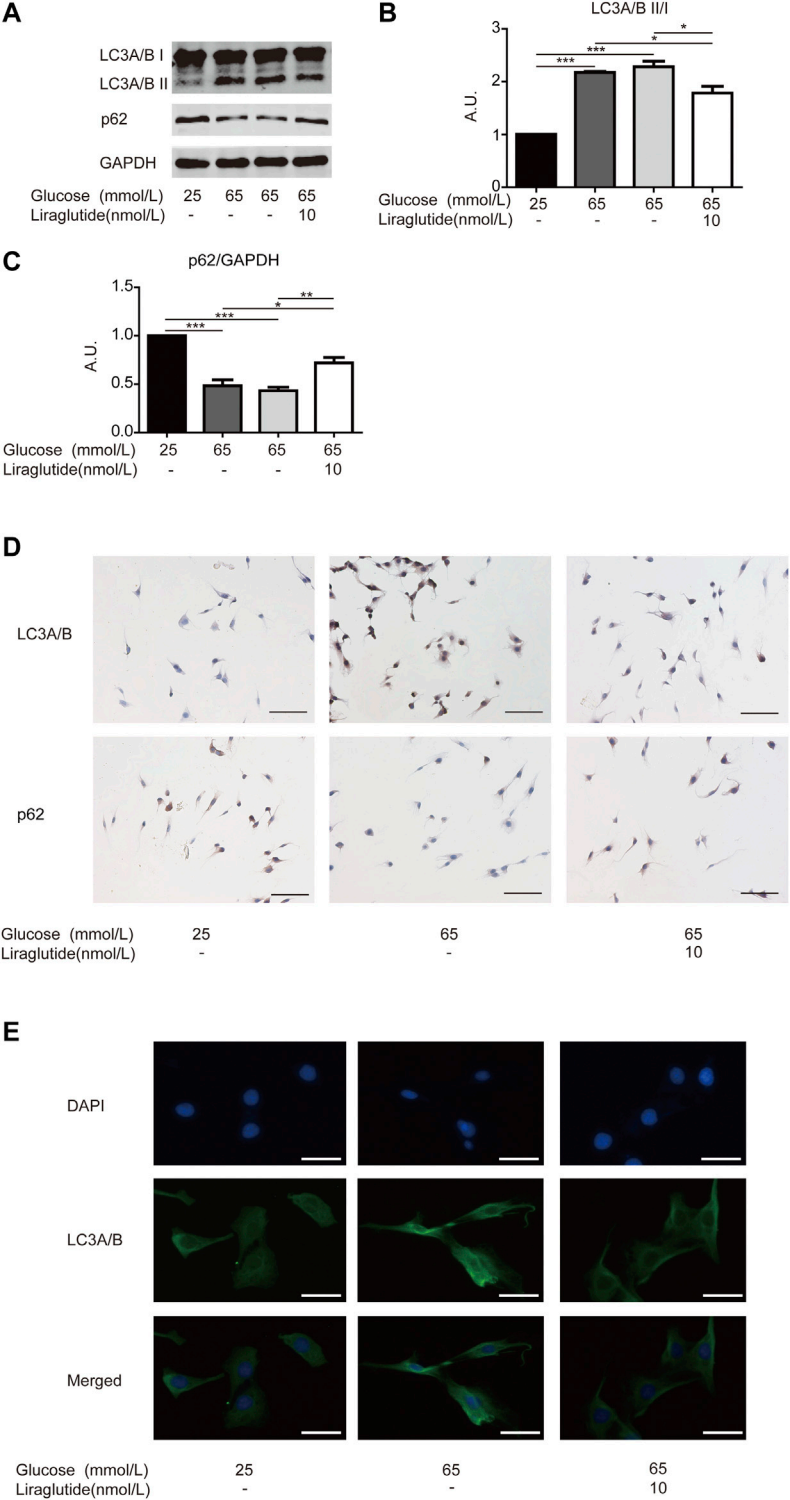
Mitophagy in RGCs was alleviated by liraglutide *in vitro*. As shown in the figure, RGCs were respectively treated with normal glucose (25 mmol/L), high glucose (65 mmol/L), high glucose (65 mmol/L) and liraglutide (10 nmol/L) for 24 h. **(A)** Relative LC3A/B and p62 expression was determined by Western blot. **(B,C)** Quantification of LC3A/B and p62 expression. **(D)** Relative LC3A/B and p62 expression was determined by immunocytochemistry. Scale bar: 100 μm. **(E)** The fluorescence expression of LC3A/B. Scale bar: 200 μm. Values are presented as mean ± SEM. *n* = 3 independent experiments. *p < 0.05, **p＜0.01, ***p < 0.001. A.U., arbitrary units.

Moreover, we measured the expression of key mitophagy proteins LC3 and p62 by Western blot and immunocytochemistry staining (Levine et al., 2011). Interestingly, high glucose upregulated the expression of LC3A/B and decreased the level of p62, whereas these changes were attenuated in cells treated with liraglutide (Figures 3A–D). Immunofluorescence results showed that the expression of LC3A/B enhanced in RGCs cultured with high glucose, but liraglutide mitigated the effect (Figure 3E) conversely.

**FIGURE 4:**
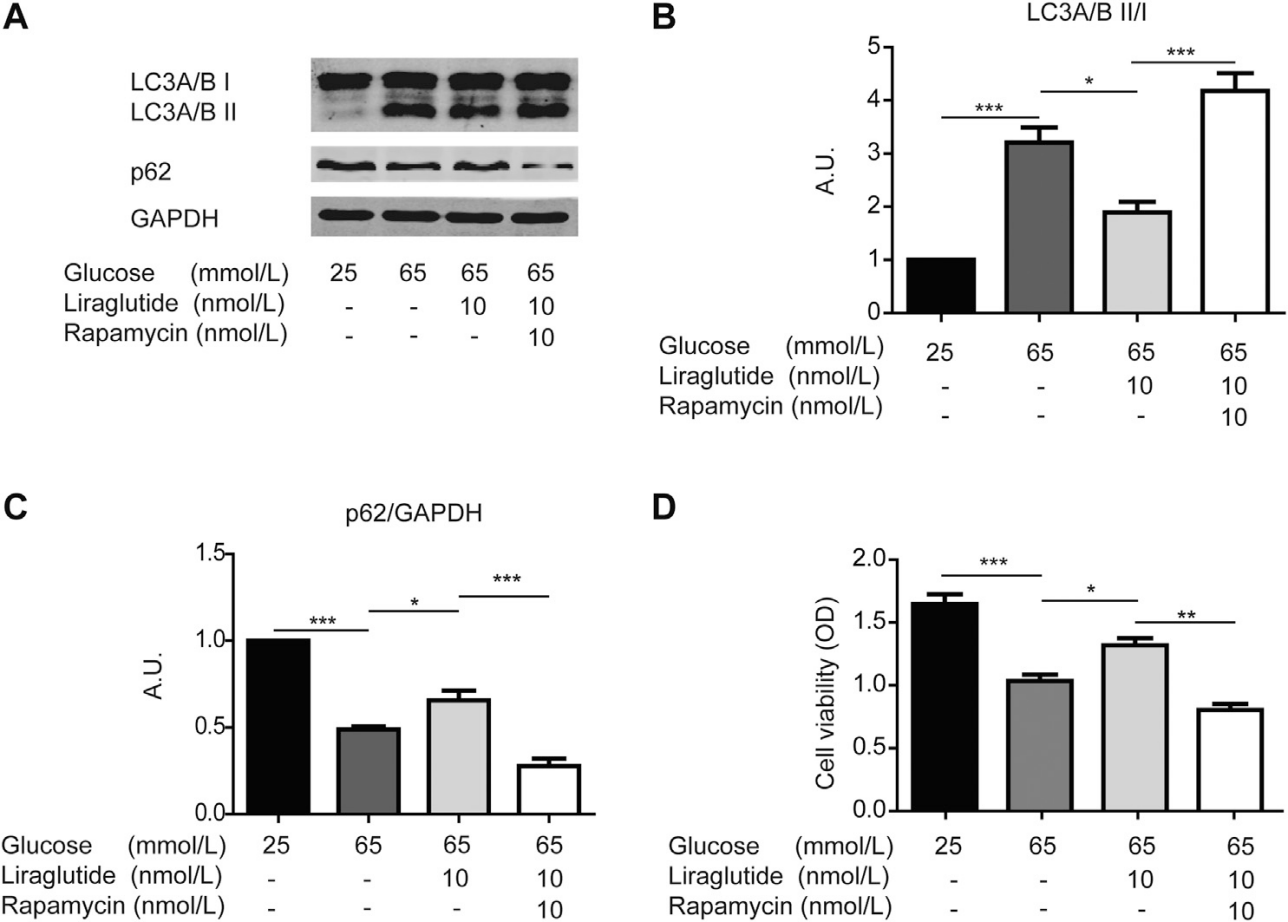
Liraglutide reduced high glucose-induced RGCs damages through mitophagy *in vitro*. As shown in the figure, RGCs were respectively treated with normal glucose (25 mmol/L), high glucose (65 mmol/L), high glucose (65 mmol/L), and liraglutide (10 nmol/L) for 24 h. Additionally, RGCs were co-treated with high glucose (65 mmol/L), liraglutide (10 nmol/L), and rapamycin for 24 h. **(A)** Relative LC3A/B and p62 expression was determined by Western blot. **(B,C)** Quantification of LC3A/B and p62. **(D)** Cell viability was detected with CCK-8 assay kits. Values are presented as mean ± SEM. *n* = 3 independent experiments. *p < 0.05, **p＜0.01, ***p < 0.001.

To further confirm the role of mitophagy, we co-incubated RGCs in a high glucose environment with liraglutide and rapamycin, a typical inducer of in the process (Lu et al., 2017). Western blot analyses showed the ratio of LC3A/B II to I increased and the level of p62 decreased after the addition of rapamycin, which indicated that mitophagy was intensified (Figures 4A–C). Meanwhile, the result of the CCK-8 detection kit showed that RGCs viability was reduced (Figure 4D). These data further illustrated that mitophagy played an important role in protective effects of liraglutide.

### Liraglutide Attenuated Mitophagy of Retinal Ganglion Cells Through PINK1/Parkin Pathway *In Vitro*

**FIGURE 5:**
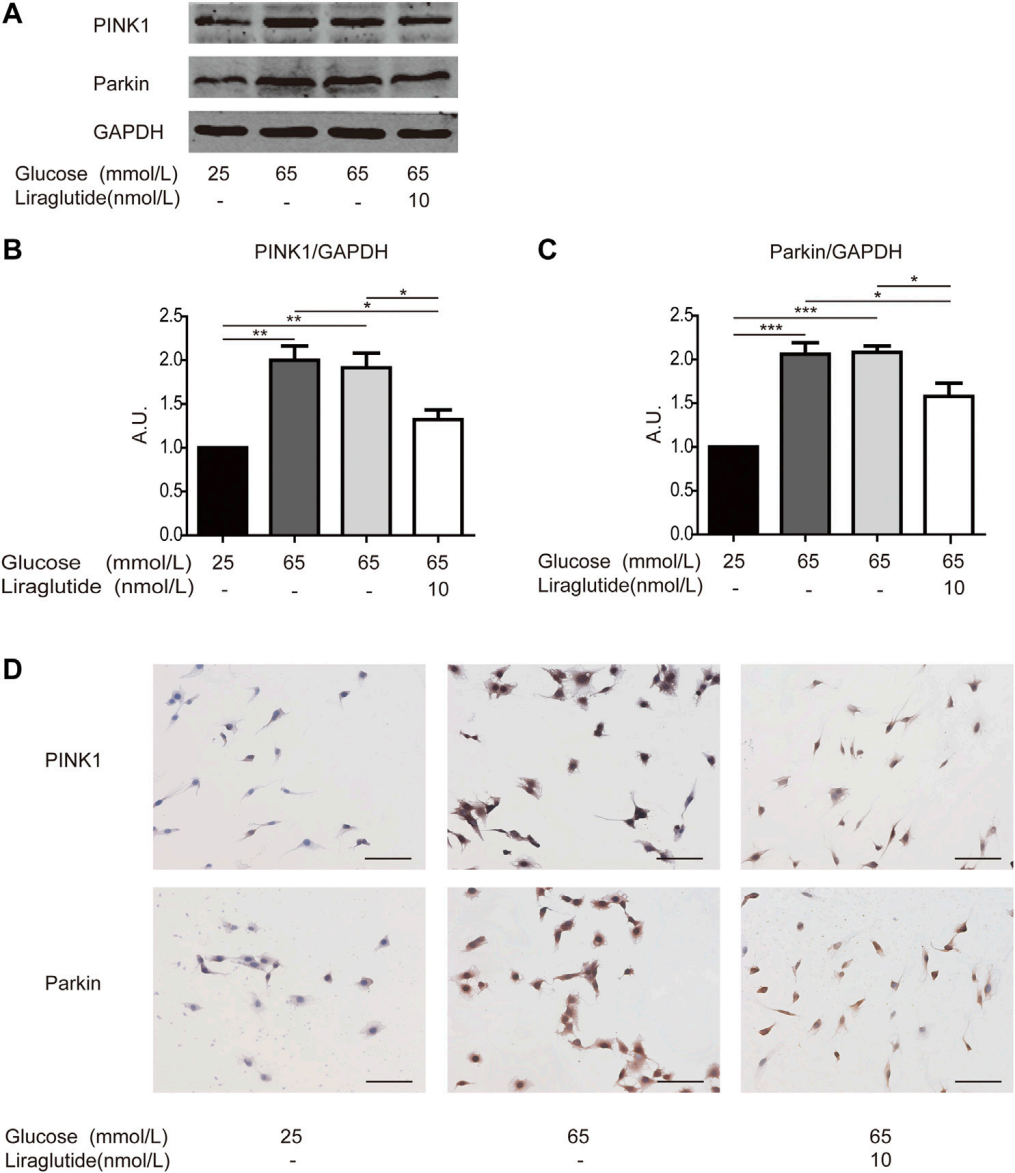
Liraglutide weakened RGCs mitophagy via PINK1/Parkin pathway *in vitro*. RGCs were respectively treated with normal glucose (25 mmol/L), high glucose (65 mmol/L), high glucose (65 mmol/L), and liraglutide (10 nmol/L) for 24 h. **(A)** Relative PINK1 and Parkin expression was determined by Western blot. **(B,C)** Quantification of PINK1 and Parkin expression. **(D)** Relative LC3A/B and p62 expression was determined by immunocytochemistry. Scale bar: 100 μm. Values are presented as mean ± SEM. *n* = 3 independent experiments. *p < 0.05, **p＜0.01, ***p < 0.001.

We subsequently studied the mechanisms in the regulation of mitophagy. We examined the expression of crucial mitophagy-related proteins, such as PINK1 and Parkin (Yoo and Jung, 2018). Western blot analyses and immunocytochemistry staining showed PINK1 and Parkin level increased in cells treated with high glucose compared with the control group, whereas the expression of PINK1 and Parkin was abated after the treatment of liraglutide (Figures 5A–D). These data demonstrated that the PINK1/Parkin pathway was involved in liraglutide-regulated prevention of mitophagy.

### Liraglutide Prevented Retinal Ganglion Cells and Mitochondrial Injuries in Diabetic Rats

**FIGURE 6:**
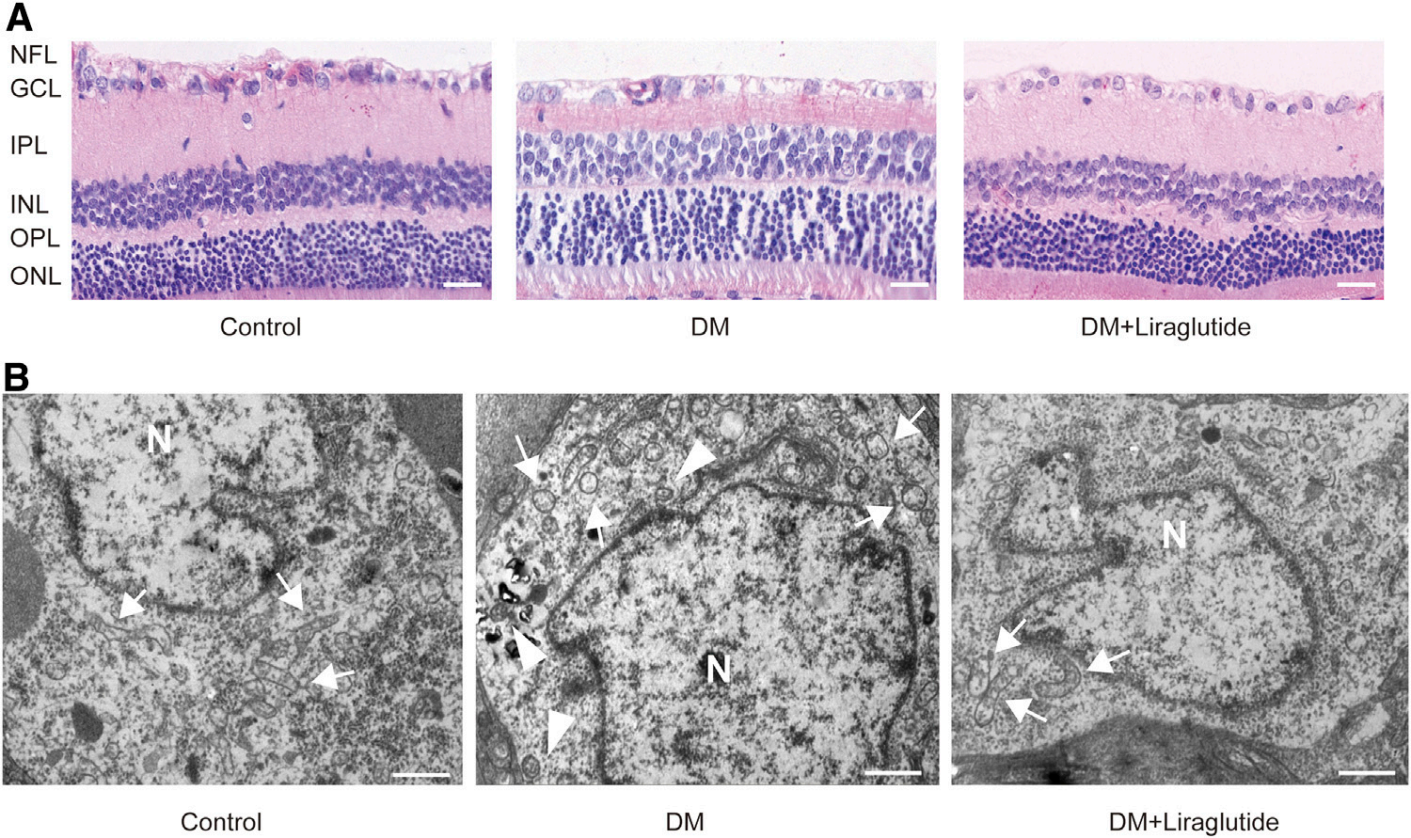
Liraglutide attenuated RGCs and mitochondrialinjuries in diabetic rats. **(A)** The beneficial effect of liraglutide on retinal morphological analysis was detected by H&E staining. Scale bar: 40 μm. **(B)** Morphology changes of RGCs were observed by TEM. Scale bar: 5.0 μm. ►, autophagosomes; , mitochondria; N, nucleus; NFL, nerve fiber layer; GCL, ganglion cell layer; IPL, inner plexiform layer; INL, inner nuclear layer; OPL, outer plexiform layer; ONL, outer nuclear layer.

In order to simulate the environment of DM *in vivo*, we established rat models of diabetes by intraperitoneal injection of STZ. Retinal ganglion cell layer (GCL) was located at the inner layer of the retina and consisted of RGCs (Hoon et al., 2014). Retinae and RGCs’ status of each group were evaluated by HE staining, respectively. The results showed that retinae of the control group were well-structured and closely arranged with regular RGC shape and deep round-like staining nuclei under microscope. However, the retinae of the DM group represented fewer cells, a disordered arrangement, and increased intercellular space. RGCs showed various shapes and sizes, vacuole-like changes, and nuclear dissolution. Interestingly, in diabetic rats treated with liraglutide, the arrangement of retinae was comparatively clear and complete. RGCs were arranged relatively neatly and showed regular forms (Figure 6A).

We next detected the ultrastructure of RGCs *in vivo* by TEM. We found RGCs of the control group had abundant organelles and normal mitochondrial structures with clearly visible mitochondrial cristae. However, in RGCs of diabetic rats, we observed swelling of mitochondria and vacuolar degeneration, and the mitochondrial cristae disappeared. In contrast, mitochondrial damage was attenuated after the treatment of liraglutide in diabetic rats. The structures of mitochondria were relatively complete and cristae was well preserved (Figure 6B). These changes were consistent with the results *in vitro*, and the results above together demonstrated that liraglutide protected RGCs in diabetic rats.

### Liraglutide Restrained Retinal Ganglion Cells Mitophagy in Diabetic Rats

**FIGURE 7:**
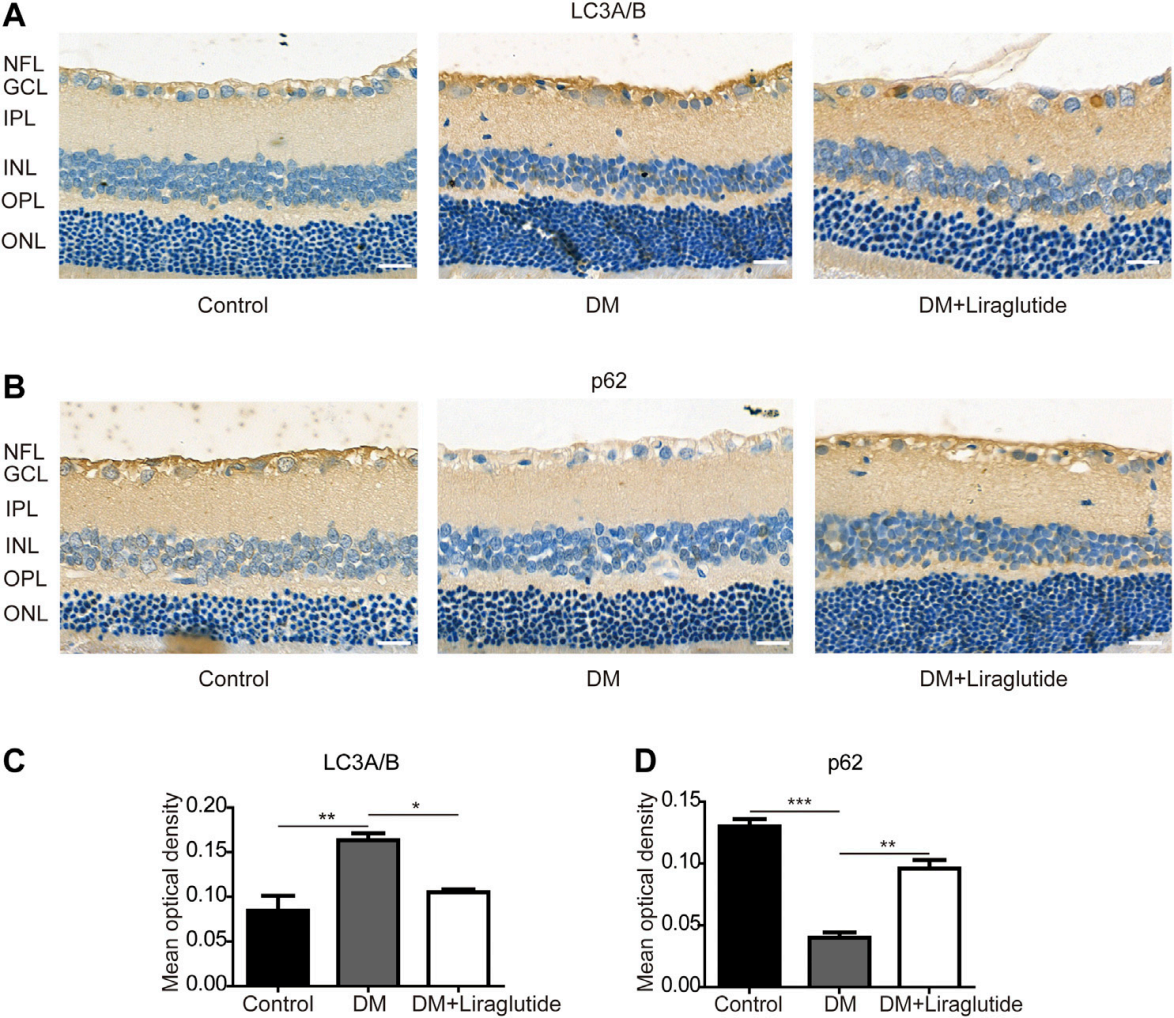
Mitophagy in RGCs was suppressed by liraglutide in diabetic rats. **(A,B)** Relative LC3A/B and p62 expression was determined by immunohistochemistry (IHC). Scale bar: 40 μm. **(C,D)** Mean optical density analysis of LC3A/B and p62 expression in RGCs in different groups. Values are presented as mean ± SEM. *n* = 5. *p < 0.05, **p＜0.01, ***p < 0.001. NFL, nerve fiber layer; GCL, ganglion cell layer; IPL, inner plexiform layer; INL, inner nuclear layer; OPL, outer plexiform layer; ONL, outer nuclear layer.

TEM was used to detect autophagosomes directly. We found autophagosomes were formed in RGCs of the DM group. However, there was almost no typical autophagosome in the liraglutide group (Figure 6B). Furthermore, we detected the same mitophagy-related proteins in rats as *in vitro* by IHC. The results showed that in GCL of diabetic rats, the expression of LC3A/B was enhanced and p62 was reduced compared with the control group, whereas these results were reversed in liraglutide treatment rats (Figures 7A–D).

### Liraglutide Regulated Mitophagy Through PINK1/Parkin Pathway in Diabetic Rats

**FIGURE 8:**
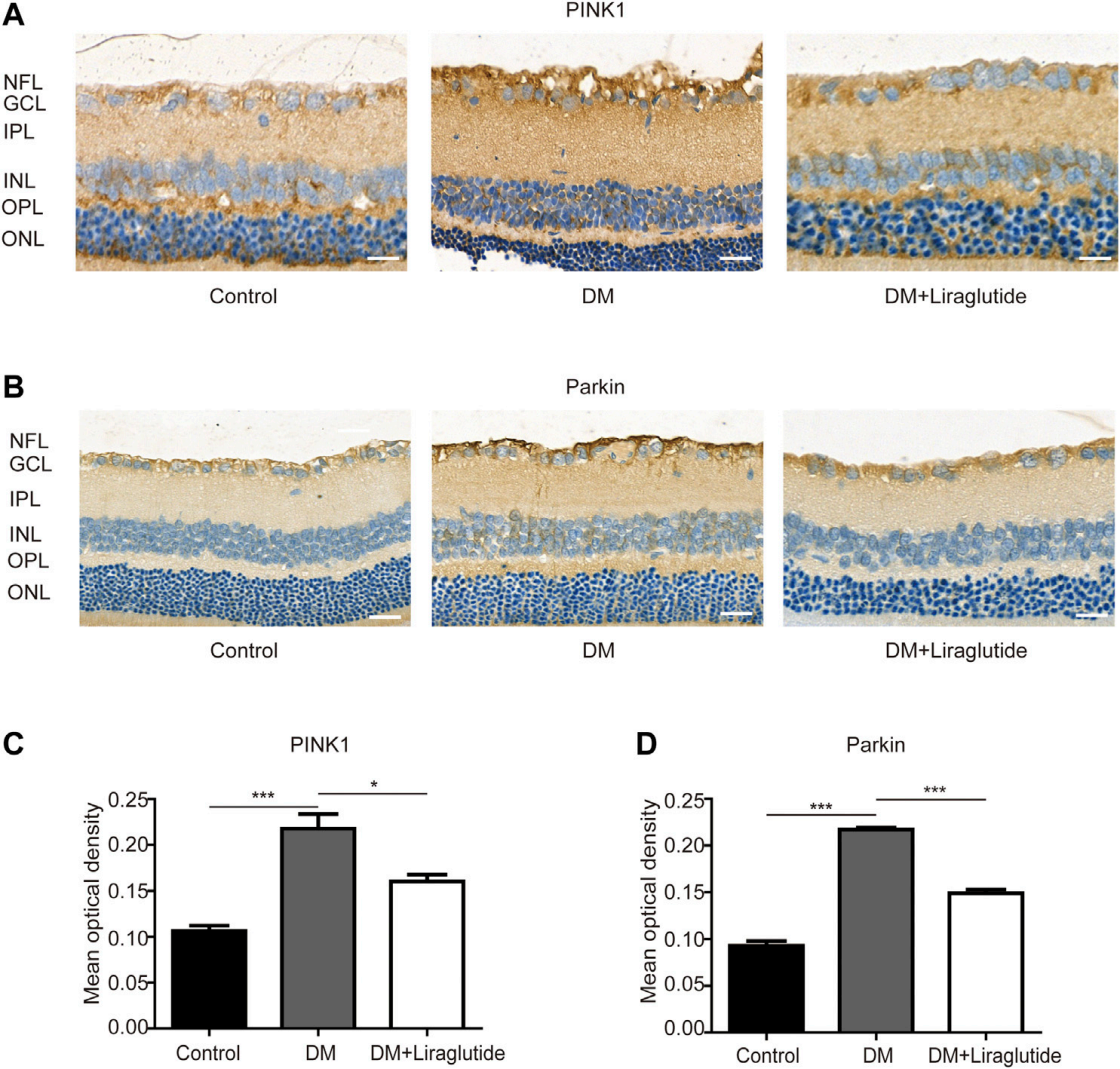
Liraglutide regulated PINK1/Parkin pathway of RGCs in diabetic rats. **(A,B)** Relative PINK1 and Parkin expression was determined by IHC. Scale bar: 40 μm. **(C,D)** Mean optical density analysis of PINK1 and Parkin expression in RGCs in different groups. Values are presented as mean ± SEM. *n* = 5. *p < 0.05, ***p < 0.001. NFL: nerve fiber layer. GCL, ganglion cell layer; IPL, inner plexiform layer; INL, inner nuclear layer; OPL, outer plexiform layer; ONL, outer nuclear layer.

We next examined the potential pathway that regulated mitophagy *in vivo* through IHC to investigate the level of PINK1 and Parkin. We found the expression of PINK1 and Parkin was increased in GCL of diabetic rats compared with the control group while reversed results were observed in liraglutide treatment (Figures 8A–D). The above results showed liraglutide inhibited mitophagy via the PINK1/Parkin pathway in diabetic rats, which was consistent with the results *in vitro*. Collectively, these data demonstrated the beneficial effect of liraglutide on RGCs in a high glucose environment, which was achieved by inhibition of the PINK1/Parkin-mediated mitophagy.

## Discussion

DR is one of the leading causes of blindness among people of working age worldwide (Klein, 2007) and may become a great challenge for healthcare systems in the future (Selvaraj et al., 2017). DR therapy still has many limitations, such as being invasive, having poor absorption, and being expensive (Selvaraj et al., 2017). It should be a consensus to protect the retina from high glucose damage when treating DM, and now the additional benefits of hypoglycemic drugs are brought into focus.

Neurodegeneration occurs early in the course of DR and promotes vascular injuries. Therefore, neuroprotective therapy may not only be beneficial to neuronal changes, but also prevent microvascular pathological changes of DR. GLP-1 analog, a commonly used clinical hypoglycemic drug, has been proven to have the ability to cross the blood-brain barrier (McClean et al., 2011; Holscher, 2012). Studies have shown that GLP-1 analog has neuroprotective properties (Hernandez et al., 2016), but its influence on DR and the potential mechanisms are not fully understood.

RGCs have long axons to transmit visual information and the metabolism of RGCs is highly active. RGC is the most common type of vulnerable cell in the retina; the injury of RGCs leads to reduction of thickness of the inner retinal layer and even loss of vision (Adamiec-Mroczek et al., 2015). Our team first found the expression of GLP-1 receptors in RGCs (Fu et al., 2012). We assumed liraglutide, a commonly used GLP-1 analog, may be advantageous to RGCs. In this study, we established a diabetic model and demonstrated the effect of liraglutide on RGCs. We found that RGCs suffered from injuries of high glucose both *in vivo* and *in vitro*, which was shown by morphological changes and cell viability. The above changes could be alleviated by liraglutide, which together suggested that GLP-1 analog could protect RGCs from high glucose conditions.

We further investigated the mechanisms of the beneficial effect of GLP-1 analog. Several studies have shown mitophagy plays a crucial role in ocular diseases (Liu et al., 2016; Miyai et al., 2019; Zhang et al., 2019). Mitophagy is one of the most important mechanisms to degrade damaged or redundant mitochondria (Um and Yun, 2017). Basal mitophagy is essential for maintaining mitochondrial homeostasis, but excessive mitophagy induces mitochondrial dysfunction, neuronal injury, and even cell death (Cherra et al., 2013; Dagda et al., 2008; Kubli and Gustafsson, 2012; Bialik et al., 2018). Mitochondrial conditions were assessed based on mitochondrial structure, ROS production, and MMP alterations. ROS are byproducts of oxidative phosphorylation and damaged mitochondria are more prone to generate ROS (Bingol and Sheng, 2016). MMP could reflect the functional metabolic status of mitochondria (Teodoro et al., 2018), and low MMP indicates the injury of mitochondria (Li et al., 2018). In our study, high glucose resulted in changes of mitochondrial morphology, which further increased ROS production and decreased MMP of RGCs. The above changes indicated that mitochondria suffered damage in such conditions. Notably, the treatment of liraglutide improved mitochondrial status both *in vivo* and *in vitro*.

Furthermore, we observed mitophagy in RGCs by detecting autophagosome and the expression of mitophagy-related proteins, such as LC3 and p62. LC3II is formed by LC3-phosphatidylethanolamine conjugate and is recruited to autophagosomal membranes (Tanida et al., 2008). P62 acts as an adaptor protein that interacts with LC3-II and is responsible for the clearance of ubiquitinated protein aggregates (Parzych and Klionsky, 2014). The results showed that the expression of LC3A/B increased and the expression of p62 decreased in the high glucose group, while the addition of liraglutide could reverse these changes. These results demonstrated that RGCs were damaged along with mitophagy increasing in a high glucose environment both *in vivo* and *in vitro*. Instead, liraglutide treatment alleviated mitophagy and RGCs viability was also regained. We further co-treated RGCs in a high glucose condition with liraglutide and rapamycin. The results showed mitophagy was enhanced and the protective effect of GLP-1 analog was weakened. These findings indicated that mitophagy was involved in the mechanisms of GLP-1 analog, protecting RGCs under high glucose condition.

Next, we studied the possible mechanisms of liraglutide-regulated mitophagy. Several mitophagy molecular pathways have been discovered in recent years. The PINK1/Parkin pathway is one of the most widely studied mechanisms of mitophagy in neurodegeneration (Chen Y. et al., 2016; Celardo et al., 2016; Truban et al., 2017; Miller and Muqit, 2019), however, the role of the PINK1/Parkin pathway in DR remains unclear. PINK1 is a mitochondrial kinase and is responsible for activation and transportation of Parkin from the cytoplasm to impaired mitochondria (Bingol and Sheng, 2016). Parkin is a cytosolic E3-ubiquitin ligase and is activated by PINK1 (Yoo and Jung, 2018). In our study, the expression of PINK1 and Parkin was increased in a high glucose condition. After the treatment of liraglutide, the level of PINK1 and Parkin was decreased. These results indicated that PINK1/Parkin pathway was involved in liraglutide-regulated mitophagy.

There are some limitations of the current study. In the high glucose condition, we proved the deleterious role of autophagy in RGCs, which could be attenuated by GLP-1 analog. Nonetheless, basic autophagy is an important way to maintain cell homeostasis (Kubli and Gustafsson, 2012; Kim and Lee, 2014) and is beneficial for the clearance of fragments and pathogens (Jiang et al., 2019). Autophagy is not simply to eliminate fragments, but a dynamic recovery system that can generate new components and energy for cell repair (Mizushima and Komatsu, 2011). Of note, defective mitophagy is related to pathological changes (Yang et al., 2014; Yan et al., 2018). Excessive mitophagy could result in the loss of energy in remnant mitochondria, insufficient mitochondrial biogenesis, and cell death, especially in cells with high demand for energy (Kubli and Gustafsson, 2012; Jiang et al., 2019). Moreover, autophagy may play a harmful role by degrading the necessary cellular matrix components (Jiang et al., 2019). Rapamycin promotes autophagy and plays a protective role in various diseases. However, rapamycin may also play a deleterious effect (Umemura et al., 2014; Carosi and Sargeant, 2019). The exact role of autophagy is not clear. Therefore, identification of mechanisms that regulate the dual functions of autophagy is meaningful in disease prevention. Furthermore, the homeostasis of mitochondrion requires the interactions of mitochondrial clearance and biogenesis (Palikaras and Tavernarakis, 2014). Mitophagy is the process of eliminating mitochondria (Guan et al., 2018). Interestingly, Agrawal et al. (2014) demonstrated that liraglutide inhibited the decrease of PGC1-α, a significant regulator of mitochondrial biogenesis. Ji et al. (2014) showed that liraglutide increased the number and area of mitochondria in diabetic models. Based on these results, it is necessary to study the effect of GLP-1 analog and mitophagy from the perspective of mitochondrial biogenesis and balance in future research. In addition, many specific molecular mechanisms are involved in mitochondrial regulation. Co-activators of the peroxisome proliferator activated receptors (PPARs) play important roles in the regulation of mitochondrial biogenesis, which could coordinate with other transcription factors, such as NRFs and ERRs (Dominy and Puigserver, 2013). Besides the PINK1/Parkin pathway, mitophagy is also mediated by BNIP3L/NIX (Schweers et al., 2007; Sandoval et al., 2008), FUNDC1 (Chen M. et al., 2016) and other signals. Intracellular molecular pathways are fraught with possibilities; exploring the protection mechanisms of GLP-1 analog from other molecular pathways in subsequent experiments may provide new ideas for the prevention and treatment of DR.

**FIGURE 9:**
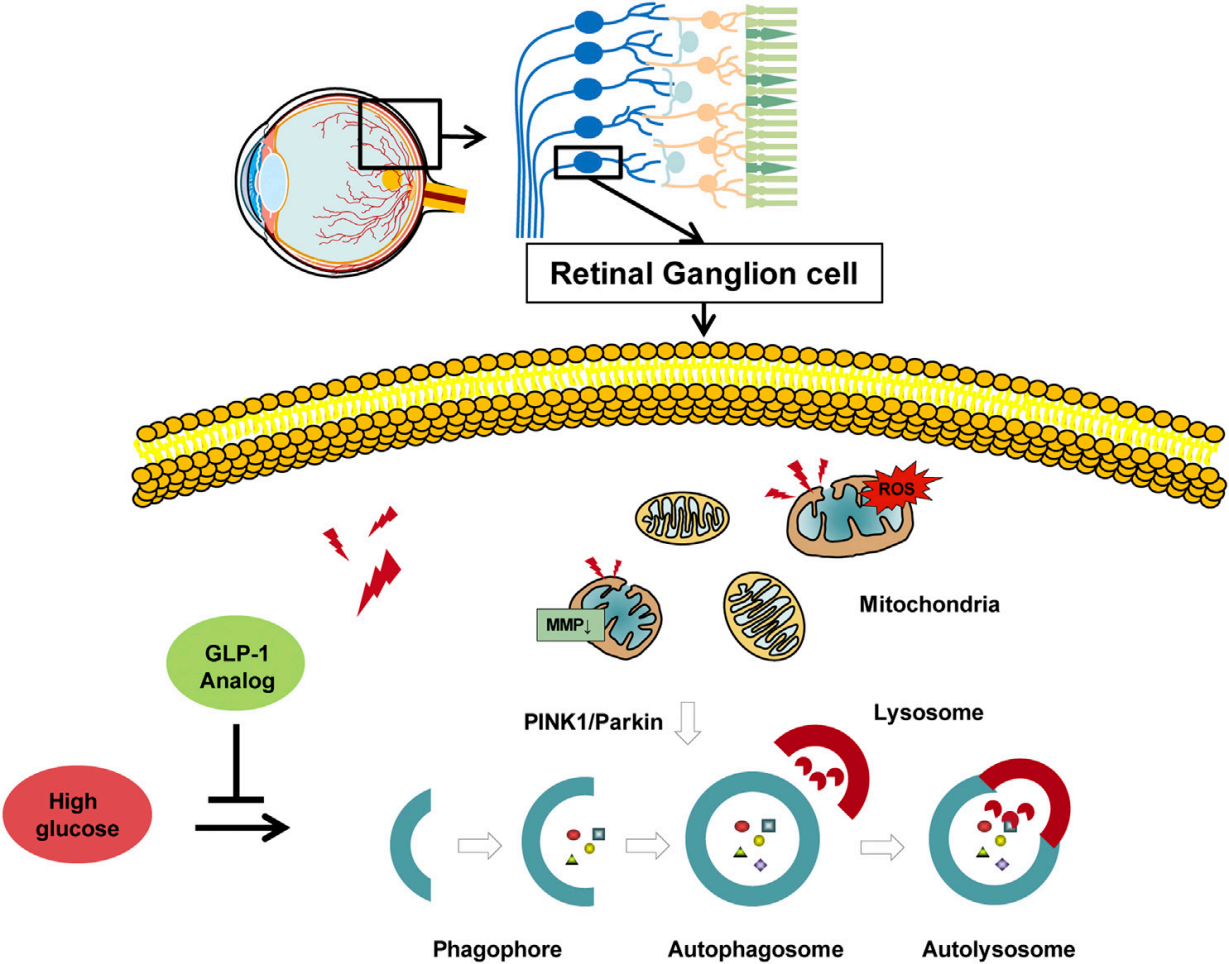
Protective role of GLP-1 analog for retinal ganglion cells via PINK1/Parkin-mediated mitophagy in diabetic retinopathy.

Together, our data provided direct experimental evidence that GLP-1 analog protected RGCs from high glucose-induced damages both *in vivo* and *in vitro*. Additionally, we examined the possible mechanisms and suggested that GLP-1 analog may mitigate mitophagy through the PINK1/Parkin pathway (Figure 9). GLP-1 analog is beneficial to retinal neurodegeneration and can be seen as a hypoglycemic drug with additional benefits on DR. We carried out the study at cellular and animal levels, which provided an empirical foundation for future clinical practice.

## Data Availability

The raw data supporting the conclusions of this article will be made available by the authors, without undue reservation.
